# The Department of Modern Nursing

**Published:** 1914-07-25

**Authors:** 


### THE DEPARTMENT OF MODERN NURSING.*

### St. Mary's Hospital.

St. Mary's, Paddington, was founded in 1845, and although added to since, has undergone no thorough reconstruction. The Clarence Wing is the most modern part of the building, which contains in all 301 beds, including thirty-four children's cots. There are special departments for eye, ear, throat, skin, teeth, and for diseases of women and children. In addition, there are special wards for therapeutic inoculation under the direction of Sir Almroth E. Wright, which form a valuable preparation for Colonial appointments. The hospital serves a large and very poor district, and the out-patient work is varied and extensive. There is a medical school. The nursing staff numbers matron and assistant, night superintendent, home sister, housekeeper, eighteen sisters, thirty-eight staff nurses, and fifty-six probationers in their first and second years.

### The First-Year Probationer.

St. Mary's has at present no preliminary school. The three months' trial period accorded to likely candidates is perforce spent in the wards, and as a proportion of failures is inevitable under this system, the time spent in breaking-in candidates who for one reason or another do not eventually go on to complete their training is a needless wear and tear on the staff. The trial three months is spent in two wards, so that the opinion of two sisters may be compared before the probationer is admitted as a regular member of the staff. These trial months count as part of the course. The number of first-year probationers varies according to the regularity with which the staff complete their full term of training. In 1910 the number of first-year probationers was twenty-six, and they made up a percentage in the staff of 22.9. In 1913 the number was thirty-four. Changes in the wards are made every three months, and throughout their course this is the length of time each probationer remains under the same sister. Three courses of lectures on nursing and elementary physiology are given to probationers during this first year, and at its close an examination takes place which decides, except under special circumstances, whether the probationer shall be retained in the service of the hospital. It is interesting to find that the plan of assigning probationers special hours for study out of their duty times has been discontinued without in any degree affecting the character of the work. It was found in practice that nurses disliked being called out of the wards for a study hour, and they apparently made little use of it, for the percentage of failures is lower since it was abolished. A good lecture and class room is reserved for the use of the nurses in training. The remuneration for this first year is £8.

### Second and Third Years.

After passing a satisfactory examination at the end of their first year the probationers enter upon the junior course, which includes lectures in elementary physiology from Sir John Broadbent and lectures on elementary physiology and surgery from Mr. Fitzwilliams. In this second year they receive £12. During their third year they receive lectures on medical nursing from Sir John Broadbent, and on surgical nursing from Mr. Fitzwilliams; and at the conclusion of these senior courses they enter for their final examination. If this is passed they are eligible to receive the hospital certificate; but it is not delivered to the nurse unless and until she completes her four years' engagement to the hospital authorities. The salary for the fourth year is £18. The size of the hospital admits of regular theatre training being afforded to all the nurses during some part of their training, the minimum period spent over this branch of work being six weeks.

The staff nurses in their fourth year receive a salary of £20. The training facilities afforded them during this final year are unique. Having passed the final examination they are eligible to receive a course of theoretical and practical massage during the winter months without extra fee. And, finally, during the last quarter of the fourth year they proceed to a three months' course in midwifery and monthly nursing at a school for midwives recognised by the Central Midwives Board. These supplementary courses of training are a generous gift on the part of the hospital, and it is satisfactory to learn that they have had the effect of raising the quality of the probationers who offer themselves as candidates. It shows a good tendency in the modern training schools that some competition is taking place on the part of the authorities to secure educated women as candidates. Instead of attempting to attract the better class of probationer by raising salaries, the wise course has been adopted at St. Mary's of offering the staff increased facilities for completing their nursing education under the ægis of the hospital.

### The Nurses' Quarters.

There is no separate Nurses' Home. The nurses' quarters are at the top of the building, and occupy the fourth and fifth floors. Under this arrangement the maximum of air and light is obtained in a locality which undoubtedly makes such commodities somewhat scarce. But it has inevitable drawbacks. There is no private entrance to the home. Nurses are compelled to use the common staircase, or deviate by another known as the Mary Stanford wing, some way round, when the staircase is thronged with patients' visitors. The lift which runs up through the building is avail-

* Previous articles of this series appeared in The Hospital of May 16, May 30, June 13, and July 11.

able for the nurses in passing from their dining-room in the basement, but not otherwise. There is not even any way of completely shutting their apartments off from the rest of the hospital, but a serving-maid is kept always on duty at the doors which give access to them. Nurses can only be permitted to receive visitors in a room reserved for this purpose on the ground floor. The bedrooms are well planned and comfortable. Some of the junior probationers occupy cubicles, two or three in a room; but these are screened off with wooden partitions, and as there is a constant moving-on process the inconvenience is not great. The sitting-room is large and pleasant; the dining-room not so good, in the basement, for the convenience of serving. Adjoining the main dining-room is a cheerful little dining-room for the sisters. Short of entire reconstruction, it is difficult to see what further steps could be taken at St. Mary's to secure the nurses' comfort. One cardinal defect is observable in the matron's quarters. The windows overlook Praed Street, with the natural consequence that when they are opened the roar of the traffic drowns all other sound. The business of the office must, therefore, be transacted in rooms cut off from ventilation, and the effect of this is apparent to the most casual visitor in the heavy air. The provision of electric fans would relieve the air considerably, and this ought not to be neglected. It is hardly possible that some injury to health should not be occasioned to those working under these conditions.

The accounts of the Nurses' Home are kept distinct under admirable secretarial organisation, and the system through which the exact cost of the nurses' table is ascertained, although all the food for the establishment is prepared in one kitchen, is well worth study. The average yearly cost per head of the nursing staff under the heads of laundry, maintenance, pensions, salaries, and uniforms is £46. The average yearly cost of the nursing per bed under the same headings is £20 4s.

### The Nurses' Hours and Recreations.

Probationers have at least two hours off duty daily, and in alternate weeks two and a half hours. Once a month they have a day and a half. Nurses on day duty get three evenings weekly from 5 p.m. to 10 p.m., with a week-end once a month. Probationers have ten days' holiday at the end of their first and second periods of six months, with three weeks in succeeding years. Sisters of two years' standing, and all holding higher nursing posts, enjoy the great advantage of an extra week's holiday in the spring.

The nurses have a good library of fiction, for which the subscription is 2s. a year; and a flourishing newspaper club, at the same subscription, whereby each sitting-room is provided with two daily papers, several weekly and nursing papers, and some monthly magazines.

In any case of serious illness nurses are warded. There is one sick room in which a nurse can be kept under observation if indisposed, for a few days, should there be no room for her in the wards. After that she would be placed in a small ward attached to the "isolation" wards.

### Pensions.

An admirable scheme is in force for encouraging nurses to belong to the Royal National Pension Fund for Nurses. One half of the annual premiums of all sisters and staff nurses is paid by the hospital while they remain in its service, the policy being for a pension of £22 10s. on the Returnable Scale, to commence at the age of fifty or upwards. Any probationer who has taken out a policy of this description during her term of training will receive back in quarterly instalments from the hospital, if appointed on the staff, one half of the premiums she has paid to the Fund during her probationership. After a nurse has been in the service of the hospital for a period of two years as sister or staff nurse, the benefit of the policy effected by the hospital on her behalf shall, if she has complied with the rules, belong to her and be assigned to her either when her pension falls due or at the date of her leaving. The premiums she pays herself are, of course, her own property.

By this well-thought-out scheme the hospital not merely gives the strongest possible encouragement to nurses to join the Pension Fund from the beginning of their nursing career, but actually gives them a present of half their premiums on condition that they remain two years in its service after the completion of the training. This adds a substantial increase to the salaries paid. By an excellent plan all money paid by the hospital into the fund and forfeited (through the failure of nurses to complete their engagements or for other causes) will remain in the Pension Fund to the credit of the hospital for the purpose of forming a Benevolent Fund to be applied at the discretion of the Board for the benefit of sisters and nurses disabled by accident or illness.

